# Hospitalizations for Pandemic (H1N1) 2009 among Maori and Pacific Islanders, New Zealand

**DOI:** 10.3201/eid1601.090994

**Published:** 2010-01

**Authors:** Ayesha Verrall, Katherine Norton, Serena Rooker, Stephen Dee, Leeanne Olsen, Chor Ee Tan, Sharon Paull, Richard Allen, Timothy K. Blackmore

**Affiliations:** Capital and Coast District Health Board, Wellington, New Zealand (A. Verrall, K. Norton, S. Rooker, L. Olsen, C.E. Tan, S. Paull, R. Allen, T.K. Blackmore); Hutt Valley District Health Board, Lower Hutt, New Zealand (S. Dee); and University of Otago, Wellington (A. Verrall)

**Keywords:** Influenza A virus, H1N1 subtype, New Zealand, hospitalization, ethnic groups, dispatch

## Abstract

Community transmission of influenza A pandemic (H1N1) 2009 was followed by high rates of hospital admissions in the Wellington region of New Zealand, particularly among Maori and Pacific Islanders. These findings may help health authorities anticipate the effects of pandemic (H1N1) 2009 in other communities.

Spread of influenza A pandemic (H1N1) 2009 virus coincided with the Southern Hemisphere winter, leading to outbreaks in Australia and New Zealand (NZ) (*1*). Quantifying the number of admissions and the age, ethnicity, and medical conditions of patients admitted to hospitals in the Wellington region because of acute illness during the first wave of pandemic (H1N1) 2009 provides valuable information to health authorities anticipating the impact of the pandemic in other regions.

## The Study

Patients suspected of having influenza and requiring hospital assessment were tested with real-time reverse transcription–PCR by using protocols from the US Centers for Disease Control and Prevention (*2*). This analysis included only persons (case-patients) with confirmed pandemic (H1N1) 2009 who were admitted to public hospital beds in the NZ Wellington and Hutt Valley regions. The results of viral testing were not available at the time of admission and therefore did not affect decisions about admission.

We identified case-patients by matching laboratory and hospital patient information systems. Sex, age, clinical service, inpatient mortality, and self-reported ethnicity were available, as was limited information about medical conditions. Ethnicity data were aggregated as NZ European, Maori, Pacific Islanders, or other (i.e., all other and unspecified ethnicities combined). Admission rates per 100,000 persons were calculated using regional denominator data from the 2006 NZ Census (*3*) and age standardized by a direct method using 10-year age groups*.*

**Figure 1:**
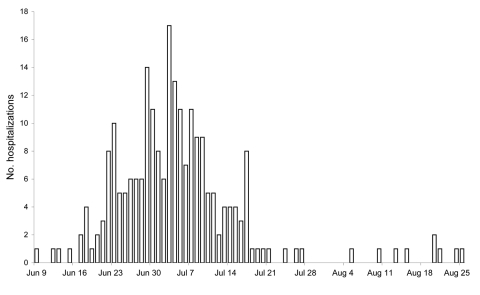
Number of hospitalizations for pandemic (H1N1) 2009, by date of admission, Wellington region, New Zealand, June–August 2009.

**Figure 2:**
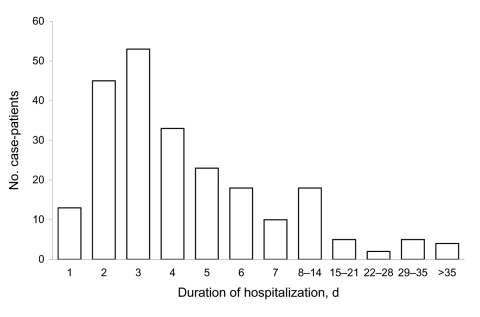
Duration of hospitalization for case-patients with pandemic (H1N1) 2009, Wellington region, New Zealand, June–August 2009.

Testing for pandemic (H1N1) 2009 began on April 24, 2009, and local community transmission was detected in the Wellington region early in June. During June 8–August 31, pandemic (H1N1) 2009 was identified in 229 hospitalized case-patients. Hospitalizations began in June, peaked in July, and then declined rapidly (Figure 1). The mean age of admitted persons was 26 years (range 0–82 years); 62% were <30 years of age. A total of 117 (51%) admissions were under adult medical services, 79 (34%), under pediatric medical services, and 15 (7%), under obstetric services. Mean duration of admission was 6.1 days (range 0–24 days) and 111 (48%) patients stayed in hospital for <72 hours (Figure 2). Nineteen (8%) case-patients were admitted to intensive care or high dependency units for at least 1 night. Five (2%) case-patients died during their hospital stay; all of these deaths were attributed to pandemic (H1N1) 2009.

Seventy-four (32%) case-patients were NZ European, 70 (31%) were Maori, 64 (28%) were Pacific Islanders, 17 (7%) were other, and 4 (2%) were unspecified. The rate of admission for NZ Europeans in the Wellington region was 25.6 per 100,000. Age-adjusted rates for Pacific Islanders and Maori were 180 and 128 per 100,000, respectively.

Ninety-one (40%) of the total group had a chronic lung condition; of these, 58 (64%) had asthma. For 116 (51%), a preexisting medical condition was described in the discharge summary. Eighteen women were either pregnant or immediately postpartum during their hospital stays. Rates of chronic respiratory conditions were lower for Pacific Islanders than for Maori or all others (28%, 41%, and 48%, respectively) (Table). Ethnicity and presence of a chronic respiratory condition were dependent (χ^2^ = 6.22, 2 df, p = 0.044). Rates of other medical conditions for Pacific Islanders, Maori, and all others were 56%, 41%, and 54% (independent χ^2^ = 3.67, 2 df, p = 0.16).

**Table:** Characteristics of 229 persons hospitalized with influenza A pandemic (H1N1) 2009 (case-patients), Wellington region, New Zealand, June–August 2009

Ethnic group	No. case-patients	Age-adjusted hospitalization rate per 100,000	Median age, y	No. (%) case-patients with chronic lung condition	No. (%)case-patients with other medical condition
Pacific Islanders	64	180	15.5	18 (28)	36 (56)
Maori	70	128	23.5	29 (41)	29 (41)
Other*	95	25.6	29	45 (47)	44 (46)

*All other and unspecified ethnicities combined.

## Conclusions

The Wellington region had higher population-adjusted hospitalization rates for pandemic (H1N1) 2009 than did New Zealand and countries overseas (*4*). In Australia, the reported rate of hospitalizations was one third that in our region (*5*). Local testing or referral patterns may have contributed to more admissions in a variety of ways. First, our rates of laboratory confirmation may be higher because of ready access to timely PCR results (*6*), facilitating investigation and case detection in persons admitted to a hospital. NZ hospitals are unlikely to have a lower threshold for admission than Australian hospitals. Although both countries have free access to hospital care for pandemic (H1N1) 2009, NZ hospitals are always busy in the winter, so clinical services attempt to manage patients in the community wherever possible.

Our study shows that Pacific Islanders and Maori were 7 and 5 times more likely, respectively, than NZ Europeans to require hospital admission. These findings are consistent with observations from previous influenza epidemics. During the 1918 pandemic, the death rate was 7-fold higher in Maori than in the NZ Europeans (*7*). In Samoa in 1918, 80% of the population was considered infected, and 7,264 died from a total population of 36,405 (*8*). Other indigenous communities, such as Aboriginals in Australia and Inuit in Canada, also appear to have higher rates of severe influenza-related illness (*9*).

Factors other than disease severity may contribute to admissions. A higher incidence of pandemic (H1N1) 2009 in Pacific Islanders or Maori households is likely to have led to greater representation across the spectrum of clinical manifestations, including severe disease (Environmental Science and Research, unpub. data). This explanation is supported by national surveillance, which demonstrated higher rates of confirmed pandemic (H1N1) 2009 in Maori and Pacific Islanders. However, this finding depends heavily on community testing, which was never uniformly available across NZ and ceased to be offered routinely once NZ switched to a manage-it phase (*10*). An alternative cause of higher rates in Pacific Islanders or Maori may be a greater readiness to self-refer to an emergency department for assessment when unwell.

This study does not address why severe influenza is more frequently observed in Pacific Islanders and Maori. We did not observe higher rates of preexisting medical conditions in the hospitalized Maori and Pacific Islanders and noted lower rates of chronic respiratory conditions in Pacific Islanders. However, other conditions were detected only on discharge summary review and not by screening or systematic questioning; thus other undocumented conditions may contribute to ethnic disparity in hospitalizations. A comparison of seropositivity rates in representative sections of high- and low-risk communities is needed to ascertain whether high hospitalization rates resulted from high rates of community transmission, preexisting conditions, or higher rates of complications of influenza. Future control strategies, including primary prevention or improved access to timely treatment, could then be refined to the needs of Maori and Pacific Islanders. In any case, targeting vaccination to the elderly and persons with known comorbid conditions will fail to protect a substantial minority of persons who need hospital care and will particularly disadvantage Pacific Islanders.

The observed age distribution is consistent with other reports describing acute pandemic (H1N1) 2009 (*11*). Most admissions were for only a few days, but a substantial minority of hospitalized case-patients required care for several weeks. Most new admissions were confined to 1 month at the start of winter.

Pandemic (H1N1) 2009 has resulted in hospital admissions and severe illness in our study population more frequently than reported elsewhere. What proportion is due to good access to nucleic acid-based testing, local disease transmission dynamics, or intrinsic susceptibility of our population to the virus is not clear. Nonetheless, our findings will interest those planning health services internationally, including other areas serving Pacific Islanders and indigenous communities.

## References

[1] World Health Organization, Regional Office for the Western Pacific. New Zealand, Australia warn Pandemic (H1N1) 2009 will stretch health system [cited 2009 Jul 23]. Available from http://www.wpro.who.int/media_centre/news/news_20090715.htm

[2] World Health Organization. CDC protocol of realtime RTPCR for influenza A(H1N1). 2009 Apr 30 [cited 2009 Jul 1]. Available from http://www.who.int/csr/resources/publications/swineflu/CDCrealtimeRTPCRprotocol_20090428.pdf

[3] Statistics New Zealand. Population estimates at 30 June 2006–2008. 2008 Dec 19 [cited 2009 Jul 1]. Available from http://www.stats.govt.nz/methods_and_services/TableBuilder/2006-census-pop-dwellings-tables/culture-and-identity/ethnic-group.aspx

[4] World Health Organization. Human infection with new influenza A (H1N1) virus: clinical observations from Mexico and other affected countries, May 2009. Wkly Epidemiol Rec. 2009;84:185–9. 19462531

[5] Department of Health and Aging. Canberra: Department of Health and Aging; 2009 [cited 2009 Sep 23]. Available from http://www.healthemergency.gov.au/internet/healthemergency/publishing.nsf/Content/C08D18DF3A0AE8D3CA257638000FA086/$File/UpdatedBulletin23rdSep12pm.pdf

[6] Faix DJ, Sherman SS, Waterman SH. Rapid-test sensitivity for novel swine-origin influenza A (H1N1) virus in humans [letter]. N Engl J Med. 2009;361:728–9. 10.1056/NEJMc0904264 19564634

[7] Rice G, Bryder L. Black November: the 1918 influenza pandemic in New Zealand. 2nd ed. Christchurch (NZ): Canterbury University Press; 2005.

[8] Influenza in Samoa. BMJ. 1919;2:499–500. 20769667 PMC2343401

[9] World Health Organization. Transcript of virtual press conference with Dick Thompson, Communications Office, and Dr Keiji Fukuda, Assistant Director-General ad Interim for Health Security and Environment, World Health Organization. 2009 Jun 9 [cited 2009 Jul 4]. Available from http://www.who.int/mediacentre/influenzaAH1N1_presstranscript_20090609.pdf

[10] Ministry of Health. Information for health professionals. Guidance on the diagnosis and management of pandemic (H1N1) 2009 in the pandemic “management” phase. Wellington (NZ): Ministry of Health; 2009 [cited 2009 Sep 23]. Available from http://www.moh.govt.nz/moh.nsf/indexmh/influenza-a-h1n1-healthsector#downloads

[11] Chowell G, Bertozzi SM, Colchero MA, Lopez-Gatell H, Alpuche-Arand C, Hernandez M, Severe respiratory disease concurrent with the circulation of H1N1 influenza. N Engl J Med. 2009;361:674–9. 10.1056/NEJMoa0904023 19564633

